# Identification and validation of SNHG gene signature to predict malignant behaviors and therapeutic responses in glioblastoma

**DOI:** 10.3389/fimmu.2022.986615

**Published:** 2022-09-08

**Authors:** Yang Fan, Zijie Gao, Jianye Xu, Huizhi Wang, Qindong Guo, Hao Xue, Rongrong Zhao, Xing Guo, Gang Li

**Affiliations:** ^1^ Department of Neurosurgery, Qilu Hospital, Cheeloo College of Medicine and Institute of Brain and Brain-Inspired Science, Shandong University, Jinan, China; ^2^ Shandong Key Laboratory of Brain Function Remodeling, Jinan, China

**Keywords:** glioblastoma, lncRNA, GSC, radiotherapy, immunotherapy, chemotherapy, pan-cancer

## Abstract

Glioblastoma (GBM) patients exhibit high mortality and recurrence rates despite multimodal therapy. Small nucleolar RNA host genes (SNHGs) are a group of long noncoding RNAs that perform a wide range of biological functions. We aimed to reveal the role of SNHGs in GBM subtypes, cell infiltration into the tumor microenvironment (TME), and stemness characteristics. SNHG interaction patterns were determined based on 25 SNHGs and systematically correlated with GBM subtypes, TME and stemness characteristics. The SNHG interaction score (SNHGscore) model was generated to quantify SNHG interaction patterns. The high SNHGscore group was characterized by a poor prognosis, the mesenchymal (MES) subtype, the infiltration of suppressive immune cells and a differentiated phenotype. Further analysis indicated that high SNHGscore was associated with a weaker response to anti-PD-1/L1 immunotherapy. Tumor cells with high SNHG scores were more sensitive to drugs targeting the EGFR and ERK-MAPK signaling pathways. Finally, we assessed SNHG interaction patterns in multiple cancers to verify their universality. This is a novel and comprehensive study that provides targeted therapeutic strategies based on SNHG interactions. Our work highlights the crosstalk and potential clinical utility of SNHG interactions in cancer therapy.

## Introduction

Glioblastoma (GBM) is the most malignant glioma in the human brain. Despite the most aggressive treatments, including surgery, radiotherapy, chemotherapy and immunotherapy, the average survival time is only approximately 14 months. A large proportion of patients will still relapse after surgery, and recurrent tumors have a higher degree of malignancy and greater resistance to radiotherapy and chemotherapy (1, 2). Traditionally, tumor progression has been considered a process involving only genetic and epigenetic changes in tumor cells. However, a large number of studies have shown that GBM tumor subtype and the microenvironment in which tumor cells grow and survive play a crucial role in tumor development (3, 4).

According to gene expression markers, GBMs can be divided into three main subtypes: proneural (PN), classical (CL) and mesenchymal (MES). Each subtype has characteristic highly expressed markers, such as *SOX2* and *OLIG2* of the PN type and *CD44* and *YKL40* of the MES type (3). In addition, MES GBM patients show a worse prognosis and greater radiation resistance than PN GBM patients (5, 6). MES GBM patients are more prone to recurrence, radiation resistance, and hypoxic necrosis (6–8). The tumor microenvironment (TME) is an integral part of tumor tissue and includes the hypoxic environment, stromal cells, macrophages, and various secretory factors (9). Through direct or indirect interactions with TME components, tumor cells cause changes in a variety of biological behaviors, such as the induction of prsoliferation and inhibition of apoptosis, angiogenesis, adaptation to hypoxia, and induction of immune tolerance. With the deepening of understanding of the complexity of the TME, increasing evidence shows that the TME plays a significant role in tumor progression, recurrence and treatment tolerance (10). Additionally, TME was implicated in the transformation of PN to the MES subtype and promote GBM progression (11).

Stemness, which is considered to indicate the potential of cells to renew and differentiate, was originally used to define the stem cells of normal mature organisms (12). Researchers now believe that there are cells that have stem cell-like characteristics in various tumors; thus, these cells can self-renew and abnormally differentiate into cells of different phenotypes, called cancer stem cells (CSCs) (13). CSC are considered to be the key factors for tumor occurrence, expansion, resistance, recurrence and metastasis and are one of the determinants of intratumoral heterogeneity (14–16). They interact with the TME to promote malignant progression (16, 17). Similarly, there are CSCs in GBM called glioma stem cells (GSCs) (18). GSCs also have a corresponding subtype corresponding to the GBM subtype, which reflects the different malignant behaviors of GSCs. Targeting GSCs has been shown to be a treatment option to improve patient survival (19).

Long noncoding RNAs are a class of noncoding RNAs with a length of more than 200 nucleotides, and an increasing number of studies have confirmed that they play a crucial role in tumor progression and therapeutic resistance (20, 21). Small nucleolar RNAs (snoRNAs), which are approximately 60-300 nucleotides in length, mainly exist in nucleoli and function as guide RNAs for the processing of transcripts (22). As the host genes of snoRNAs, long noncoding small nucleolar host genes (SNHGs) are involved in the development of various cancers, and their role is independent of snoRNAs; SNHGs are mainly involved in tumorigenesis, apoptosis, tolerance to radiotherapy and chemotherapy, and survival (23, 24). Previous studies have shown that *SNHG1*, *SNHG3*, *SNHG4*, *SNHG6*, *SNHG7*, *SNHG12*, *SNHG14*, *SNHG16*, *SNHG17*, *SNHG20* and *SNHG22* promote tumor growth as oncogenes, while *GAS5* and *SNHG9* act as tumor suppressor genes. In addition, *SNHG5*, *DANCR*, and *SNHG15* play dual roles and thus have attracted the attention of many scholars (25–28). However, due to technical limitations and the fact that the occurrence and development of tumors is characterized by the interaction of numerous tumor-related factors in complex ways, the above studies are limited to one or two SNHGs and cell types. At present, the relationship between SNHG family genes and GBM subtype, TME and stemness features is not clear. Therefore, a comprehensive understanding of how SNHGs regulates these three characteristics will help us deepen our knowledge of the occurrence and treatment of GBM.

In this study, genomic information for GBM patients from The Cancer Genome Atlas (TCGA) was integrated to comprehensively evaluate SNHG interactions. Additionally, we screened *SNHG18* to verify its effect on the self-renewal ability and subtypes of GSCs. We revealed two distinct patterns of SNHG interactions, and surprisingly, the subtype characteristics underlying these two patterns were highly consistent with the PN and MES subtype, suggesting that SNHG interactions play a significant role in shaping GBM subtypes. In addition, these two patterns were implicated in immune cell infiltration and stem cell features. Additionally, we established a scoring system to quantify the SNHG interaction model of individual patients, further verified the role of the SNHG interaction model in the response to anti-PD-1/L1 immunotherapy, radiotherapy, chemotherapy, and screened AZD3759 to verify its therapeutic effect on GSC.

## Materials and methods

### Data collection and analysis

Gene expression profiling data and clinical information for patients providing GBM and normal tissues were downloaded from the TCGA database (https://cancergenome.nih.gov/). Several GBM cohorts were enrolled in this study: the Chinese Glioma Genome Atlas (CGGA) cohort (https://www.cgga.org.cn/), Rembrandt cohort, Gravendeel cohort, Frejie cohort, and Murat cohort. Two cohorts of immunotherapy-treated patients were eventually included in this study: patients with advanced uremic tumors treated with atezolizumab (IMvigor210 cohort) and patients with metastatic melanoma treated with pembrolizumab (GSE78220 cohort). The expression data and detailed clinical traits for these cohorts were obtained from the http://research-pub.Gene.com/imvigor210corebiologies and Gene Expression Omnibus (GEO, http://www.ncbi.nlm.nih.gov/geo/), respectively. The drug sensitivity data of diverse cell lines were downloaded from the Genomics of Drug Sensitivity in Cancer (GDSC, www.cancerRxgene.org) dataset. Corresponding cell line expression data were obtained from the Cancer Cell Line Encyclopedia (CCLE, https://portals.broadinstitute.org/ccle/) dataset. Pan-cancer RNA sequencing data, somatic mutation data and clinical information were downloaded from the UCSC Xena data portal (https://xena.ucsc.edu/).

### Machine learning downscaling

Twenty-five SNHGs were used as candidates entered into the least absolute shrinkage and selection operator (LASSO) regression model. Some candidate SNHGs were completely ignored in the evaluation of the output. For the remaining five SNHGs, logistic regression analysis, classification tree analysis and random forest algorithms were applied to determine the weights of each gene.

### Cell lines and reagents

All patient-derived GSC cell lines and neural progenitor cells (NPCs) were kindly donated by Dr. Krishna P.L. Bhat (The University of Texas, M.D. Anderson Cancer Center, Houston, TX). GSC11, GSC8–11, GSC20, GSC267, GSC28 were established and widely applied in previous studies (6, 29, 30); their subtypes had already been identified according to the metagene score for PN or MES subtypes based on Philips and Verhaak gene set, respectively (3, 5). All cell lines were cultured in medium prepared from DMEM/F12 (10565018; Gibco, USA), 2% B-27 no serum supplement (17,504,044; Gibco, USA), 20 ng/mL human recombinant EGF (236-EG; R&D Systems, USA), and 20 ng/mL human recombinant bFGF (233-FB; R&D Systems) using a 37°C, 5% CO2 environment. Accutase solution (A6964; Sigma–Aldrich, USA)-digested tumor spheres were used for passaging. All cell lines used in the experiments were free of mycoplasma contamination. Poly-L-ornithine solution (P4957; Sigma–Aldrich) and laminin (L4544; Sigma–Aldrich) were used to coat the plates to make the cells adhere to the wall for the experiment.

### Cell transfection and dosing

Small interfering RNAs (siRNAs) (Genepharma, Shanghai, China), Short hairpin RNAs (shRNAs) (Genepharma, Shanghai, China) and a Lipofectamine 3000 kit (Invitrogen, Carlsbad, CA, USA) were used to transfect GSCs for loss-of-function experiments. The siRNA sequences are detailed in **Table S8**. The shRNA plasmids were selected and inserted into the pLVX-IRES-Puro vector for stable knockdown, with empty plasmid used as a control. The shRNA sequences were constructed according to siRNA. AZD3759 (synonyms: zorifertinib, C_22_H_23_ClFN_5_O_3_) was purchased from MCE (https://www.medchemexpress.cn/), dissolved in DMSO and diluted in DMEM/FBS to a final drug concentration of 50 μM for *in vitro* experiments.

### RNA extraction and quantitative real-time PCR

TRIzol (Invitrogen, USA) was used to extract total RNA according to the manufacturer’s protocol. Reverse transcription was performed using a high-capacity cDNA reverse transcription kit (Toyobo, FSQ-101, Shanghai, China) according to the manufacturer’s protocol. qRT–PCR was performed using the Mx-3000P quantitative PCR system (Applied Biosystems, Foster City, USA). Relative expression levels were calculated using the 2^-ΔΔCT^ method. The sequences of the primers are listed in **Table S8**.

### Neurosphere formation assay

GSCs were seeded in 6-well plates at 1000 cells per well. After 1 to 2 weeks of incubation in GSC culture medium, images were obtained by microscopy, and sphere diameters were measured using ImageJ for quantitative analysis.

### Extreme limiting dilution assay

GSCs were seeded into 96-well plates with a density gradient of 1, 2, 4, 8, 16, 32, 64 and 128 cells per well in 10 replicates. The number of wells with successfully formed tumor spheres was counted 7-14 days after implantation. The data were analyzed using ELDA software (http://bioinf.wehi.edu.au/software/elda/).

### Immunofluorescence assay and antibodies

Cells were fixed with 4% paraformaldehyde for 30 min and treated with 0.3% Triton X100 in PBS for 7 min. Then, the cells were blocked with 5% BSA for 60 min. Then, the cells were incubated with primary antibody overnight at 4°C and washed three times with PBS. Cells were incubated with DAPI for 30 min. The images were observed using a LeicaSP8 confocal microscope (Leica Microsystems, Wetzlar, Germany). The following primary antibodies were used: γ-H2AX (9718; Cell Signaling Technology; 1:400), CD44 (3570; Cell Signaling Technology; 1:400), and SOX2 (3579; Cell Signaling Technology; 1:400).

### Comet assay

Cells were diluted in PBS at a density of 3*10 (6) cells/ml. Cell suspensions were mixed with low-melting point agarose (Sigma) and transferred to precoated slides. Cells were lysed in alkaline lysis solution for 24 h at 4°C. Slides were washed with alkaline electrophoresis buffer and electrophoresed at 25 V for 30 min. After washing in dH2O, the nuclei were treated with 70% alcohol for 5 min, stained with SYBR Green dye for 20 min, and washed again. The representative images were captured leveraging a fluorescence microscopy.

### Xenograft model and treatments

We constructed GSC267 cells labeled with luciferase (GSC267-luciferase) *via* lentiviral transfection. All animal experiments were performed with approval from the guidelines of the Institutional Animal Care and Use Committee of Qilu Hospital of Shandong University. Four-week-old male BALB/c nude mice (SLAC Laboratory Animal Center; Shanghai, China) were bred under specific pathogen-free conditions at 24°C on a 12-h day-night cycle, preparing for the establishment of an intracranial GSC *in situ* growth model. We randomly divided the animals housed under similar conditions into control and experimental groups. 5 × 10 (5) GSC267-luciferase cells were injected intracranially into the mice. When irradiation was necessary in animal studies, tumor-bearing mice were given four doses of IR (2.5 Gy each) within 8 to 12 days after implantation. In the dosing group, PBS or an equal volume of AZD3759 (15 mg/kg) was injected daily in the tail vein 7 days after GSC implantation. The tumor progression *in vivo* was measured by bioluminescence after intraperitoneal injection of 150 mg/kg luciferin; the signal was detected, and images were taken with an IVIS Lumina series III *ex vivo* imaging system (PerkinElmer, USA).

### RNA sequencing of human tumor tissue

We obtained tumor samples from 12 patients who were treated for glioma at Qilu Hospital, Shandong University. Total RNA from tissues was isolated by using TRIzol Reagent (Invitrogen) according to the manufacturer’s instructions. The detailed information of RNA quantification and qualification, library preparation, quality control etc. were described in the Supplementary Materials and Methods.

### Unsupervised clustering of SNHGs

Expression data for 25 SNHGs were extracted from the TCGA database. The ConsensusClusterPlus package was used to perform unsupervised clustering analysis. GBM patients were classified into different clusters for further analysis, and a consensus clustering algorithm was used to determine the exact number of clusters.

### Gene set variation analysis

The gene sets “c2.cp.kegg.v7.4” and “c5.go.bp.v7.4” were acquired from the Molecular Signatures Database (MSigDB) v7.4 (http://www.gsea-msigdb.org/gsea/msigdb/index.jsp). The “GSVA” R package was applied to conduct GSVA. A P value less than 0.05 indicated that the difference was statistically significant.

### Calculation of stemness indices

We trained the stemness index model on embryonic expression data obtained from the Progenitor Cell Biology Consortium (PCBC, https://progenitorcells.org/frontpage) dataset. Then, we applied the calculation model to GBM patients to qualify the stemness indices according to a one-class logistic regression (OCLR) algorithm.

### Estimation of TME cell infiltration

Single-sample gene set enrichment analysis (ssGSEA) was used to assess the relative immune cell infiltration according to 28 immune-related gene signatures obtained from the dataset of Bindea et al. The CIBERSORT algorithm was used to quantify the level of infiltration of 22 different immune cells among pan-cancer analysis.

### Identification of differentially expressed genes

DEGs among two SNHG clusters were identified based on the limma package in R software. The p value< 0.05 were considered significant criteria.

### Generation of the SNHG scoring system

First, we performed univariate Cox regression analysis to calculate the prognostic value of each DEG (**Table S3**). The DEGs with a p value< 0.05 were extracted to construct the SNHG signature. Then, principal component analysis (PCA) was performed to construct an SNHG score model. Both PC1 and PC2 were extracted to generate a scoring system. The formula was as follows:


$$\begin{array}{l} SNHscore=\sum (-PC1_{i}-PC2_{i}) \end{array}$$

where i represents the expression of SNHG cluster-related genes.

### Association analysis of the SNHG score and drug sensitivity

We obtained the transcription profile data as well as drug information (the AUC value and targeted pathways of drugs in diverse cell lines) from the CCLE and GDSC databases, respectively. Then, Spearman correlation analysis of the SNHG score and AUC value was performed to identify the potential drugs related to the SNHG score.

### Statistical analysis

R 4.01 (https://www.R-project.org) was used to analyze and visualize all statistical data. One-way ANOVA and Kruskal–Wallis tests were used to make comparisons of differences between three or more groups. To analyze the correlation of patient survival and SNHG score, we classified patients into low and high SNHG score groups according to the cutoff point determined by the survminer R package. The Kaplan–Meier method was used to generate survival curves, and the log-rank test was applied to perform significance tests. A receiver operating characteristic (ROC) curve was utilized to evaluate the specialty and sensitivity of the SNHG score. The pROC R package was used to qualify the area under the curve (AUC) value. All statistical P values are two-sided, and P<0.05 was considered to indicate a statistically significant difference.

## Results

### Landscape of the clinical features of SNHGs in GBM

**Figure 1:**
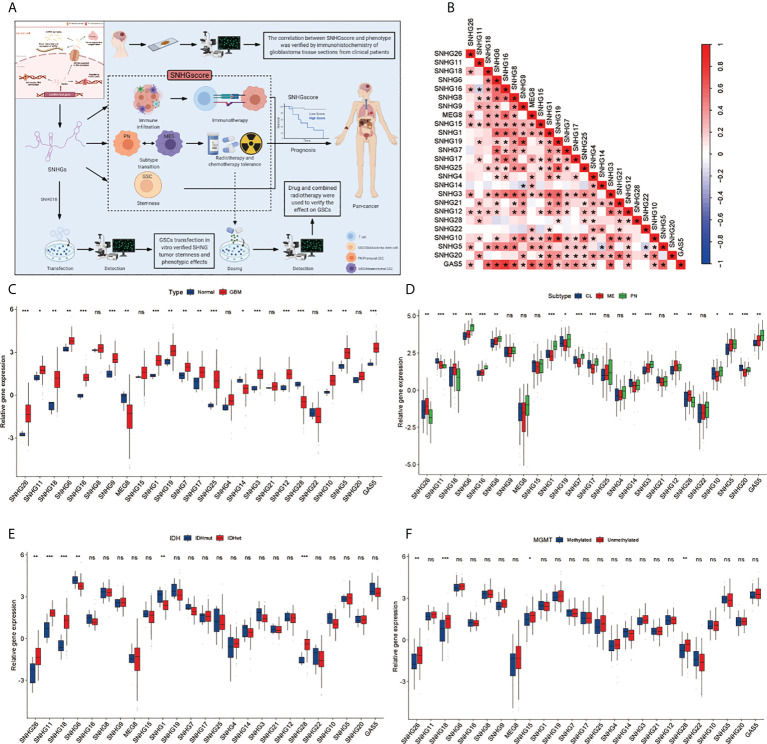
Landscape of Clinical features of SHNGs in GBM. **(A)** Article research ideas and several main mechanisms of SNHG in cancer cells. **(B)** The correlations between the 25 SNHGs were calculated in GBM using the Spearman correlation analysis. (*P<0.05). **(C)** The expression of 25 SNHGs between normal and GBM tissues in TCGA. **(D)** The expression of 25 SNHGs between TCGA GBM subtypes. **(E)** The expression of 25 SNHGs between IDH mutant and IDH wild subtypes in TCGA. **(F)** The expression of 25 SNHGs between methylated modification and unmethylated modification. All data are presented as the means ± SD, ns, P > 0.05, *P < 0.05, **P < 0.01, ***P < 0.001.

A total of 25 SNHGs were finally identified in this study. **Figure 1A** summarizes the common nuclear and cytoplasmic effects of SNHGs in tumor cells and the landscape of this study. **Figure S1A** illustrates the overall flow of this study. We first used Spearman correlation analysis to calculate the correlation of 25 SNHGs in GBM (**Figure 1B** and **Table S1**). We observed that the expression levels of SNHG family members were mostly positively correlated. The expression of most SNHGs was higher in GBM tissues in comparison with normal tissues, whereas *SNHG14*, *SNHG28* and *MEG8* exhibited higher expression in normal tissues than in GBM tissues (**Figure 1C**). Additionally, *SNHG5*, *SNHG11*, *SNHG12*, *SNHG14*, *SNHG18*, *SNHG26*, and *SNHG28* showed higher expression in the MES subtype than in the PN subtype (**Figure 1D**). In terms of IDH mutations, *SNHG11*, *SNHG18*, *SNHG28*, and *SNHG26* were highly expressed in IDH wild-type samples (**Figure 1E**). *SNHG15*, *SNHG18*, *SNHG26*, and *SNHG28* showed higher correlations with unmethylated MGMT expression (**Figure 1F**). In the same way, a total of 17 SNHGs were analyzed for IDH status and GBM subtype in the CCGA database. MEG8, SNHG11 and SNHG18 were more expressed in GBM patients with IDH wild type than in patients with IDH mutation (**Figure S2A**). The expression of GAS5, SNHG1, SNHG3, SNHG12, SNHG15, SNHG16 and SNHG18 was significantly upregulated in MES subtype GBM patients (**Figure S2B**). The above analysis showed that SNHGs are mainly cancer-related, and most of them are significantly differentially expressed in the MES and PN subtypes, suggesting that the imbalance in the expression of SNHGs plays a significant role in the occurrence, progression and subtype determination of GBM.

### Screening and validation of SNHG18 in GSCs

**Figure 2:**
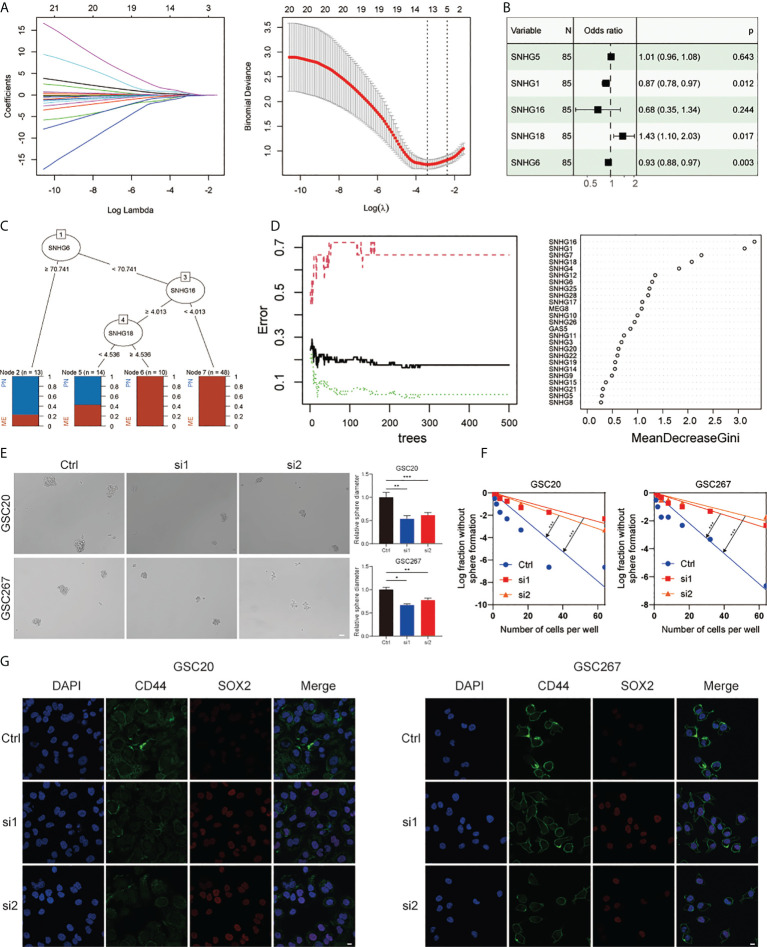
The role of SNHG18 among the SNHG family in GSCs. **(A)** LASSO coefficient profiles of 25 SNHGs and the optimal penalization coefficient (λ) via 10-fold cross validation based on partial likelihood deviance. **(B)** Multiple logistic regression analysis of the remaining five SNHGs selected by LASSO. **(C)** A classification tree was built to optimize the GBM subtype stratification. **(D)** Random-forest algorithm was utilized to screen for the most important SNHG correlated with GBM subtype. ntree: number of decision trees. **(E)** Cell spheres formation assay of GSC20 as well as GSC267 transfected with si-SHNG18, or si-Ctrl and column plot represented the relative spheres diameter (scale bar=50 μm). **(F)** Limiting dilution assay for GSC20 as well as GSC267 transfected with si-SHNG18 or si-Ctrl. **(G)** IF assay exhibited the level of CD44 and SOX2 in GSC20 and GSC267 transfected with si-SHNG18 or si-Ctrl (scale bar=15 μm). All data are presented as the means ± SD, ns, P > 0.05, *P < 0.05, **P < 0.01, ***P < 0.001.

To verify the importance of SNHGs in GBM at the experimental level, we identified the five best candidate SNHGs using the LASSO algorithm (**Figure 2A**). Next, we further analyzed the role of five SNHGs in PN and MES subtypes by applying logistic regression and classification tree and random forest algorithms. Finally, we selected *SNHG18* as a representative gene based on the above analysis (**Figures 2B–D**). First, we performed q-PCR to assess the basal expression of SNHG18. As shown in **Figure S3B**, the expression of *SNHG18* in MES GSCs was significantly higher than that in PN GSCs and NPCs. We further performed neurosphere formation assay and ELDA after knockdown of SNHG18 (**Figure S3C**). We observed that knockdown of *SNHG18* in GSCs resulted in a significant inhibition of tumorsphere expansion (**Figure 2E**) and reduced sphere formation ability (**Figure 2F**). Subsequently, we performed IF to evaluate the effect of *SNHG18* on GBM subtypes (**Figure 2G**). Knockdown of *SNHG18* resulted in a significant decrease in the MES marker CD44 and increase in the PN marker SOX2. Finally, knockdown of SNHG18 significantly reduced the tumorigenicity of GSCs and prolonged the survival of mice *in vivo* (**Figures S3D, E**). Collectively, we reveal that the representative gene *SNHG18* plays an important role in the tumorigenesis and subtype determination of GBM and GSCs.

### Interaction patterns of 25 SNHGs

**Figure 3:**
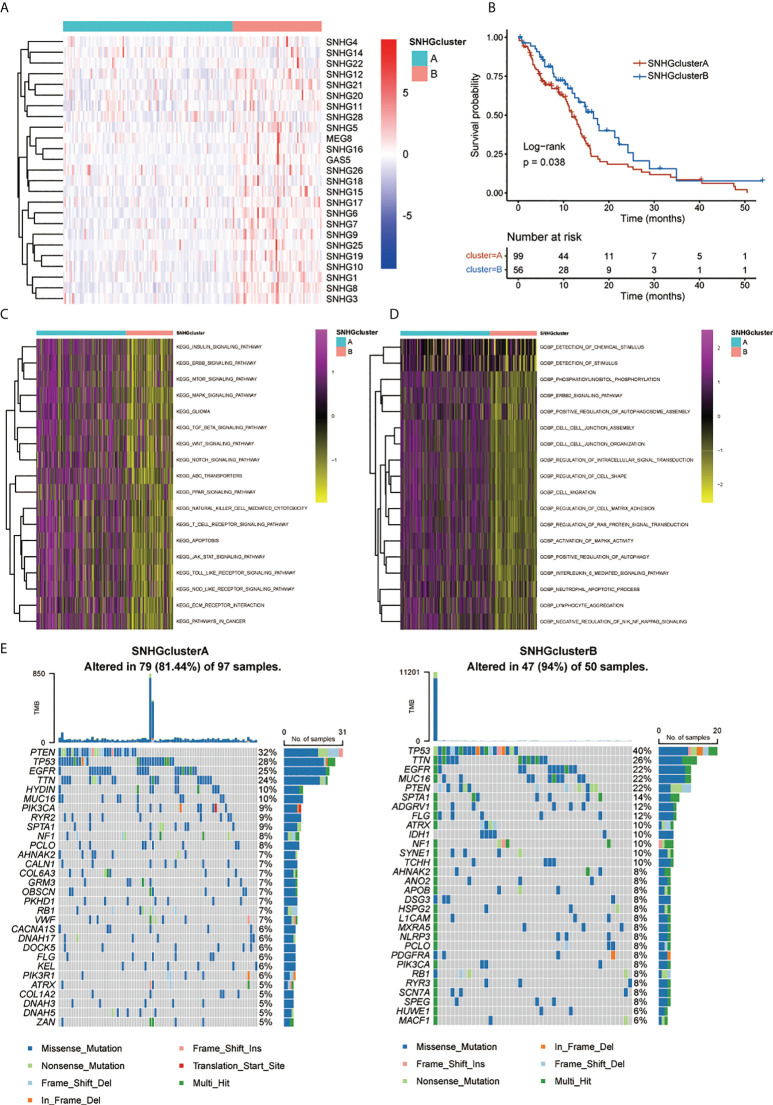
Patterns of SNHGs interaction and relative biological features. **(A)** Heatmap showed expression of 25 SNHGs and clusters in the TCGA GBM samples. **(B)** Survival analyses for high SNHGclusterA and SNHGclusterB patients using Kaplan-Meier (KM) curves (P = 0.038, Log-rank test). **(C, D)** GSVA analysis exhibited the enrichment level of diverse biological pathways for different SNHG interaction patterns. c Utilize KEGG genesets; d Utilize GO genesets. **(E)** The waterfall plot presented the mutations in the top 30 genes among SNHGclusterA and SNHGclusterB.

A univariate Cox regression model revealed the prognostic values of 25 SNHGs in TCGA and 17 SNHGs in CGGA for GBM patients (**Figures S1C, S2C, S3A**). The comprehensive landscape of SNHG interactions, gene connections and their prognostic significance for GBM patients was depicted using a network (**Figure S1B**). Then we classified patients with qualitatively different SNHG interaction patterns based on the expression of 25 SNHGs, and two distinct interaction patterns were eventually identified. We termed these patterns SNHGclusterA and SNHGclusterB (**Figure 3A**, **Figures S4A, B**). Prognostic analysis for the two SNHG clusters revealed a marked survival advantage for patients with the SNHGclusterB interaction pattern (**Figure 3B**). To explore the biological behavior between SNHG interaction clusters, we performed GSVA. In comparison with SNHGclusterB, SNHGclusterA was enriched in cancer-related pathways such as ERBB, mTOR and MAPK; immune-related pathways such as TOLL-like and NOD-like pathways etc. using KEGG signatures (**Figure 3C**). SNHGclusterA was enriched in the MAPK and ERBB pathways and implicated in various features of invasion and migration such as cell migration, cell matrix adhesion etc. using GO signatures (**Figure 3D**). We then summarized the tumor somatic mutation rates of the two clusters and observed that SNHGclusterA had a higher PETN mutation with a 32% mutation rate, while SNHGclusterB had a higher TP53 mutation rate than A (**Figure 3E**).

### Correlation of SNHG pattern with GBM subtype, TME and stemness

**Figure 4:**
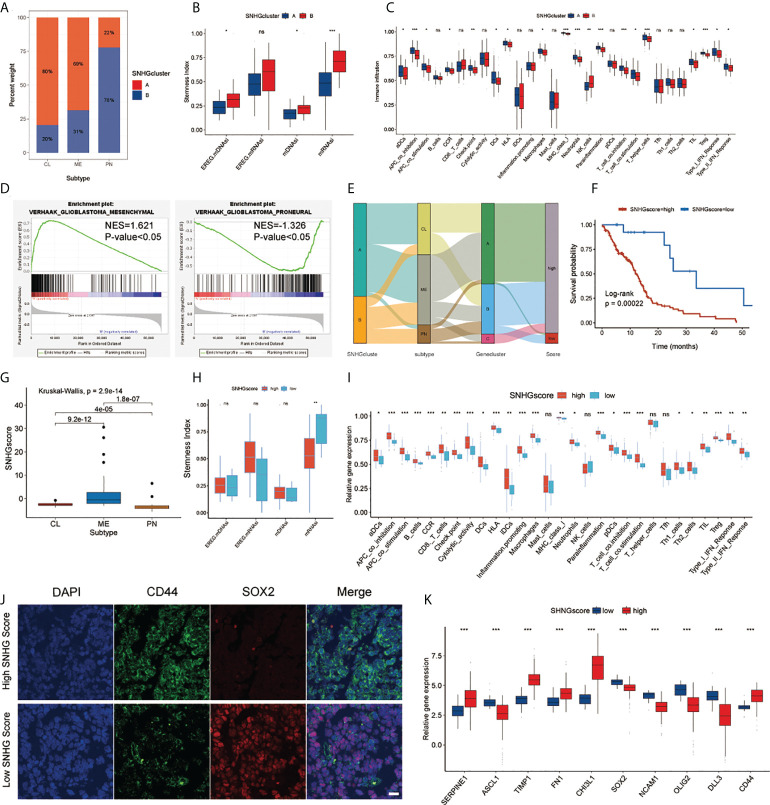
Analysis of SNHG interaction pattern and generation of SNHGscore. **(A)** The proportion of two interaction patterns in the three GBM subtypes. **(B)** Differences in stemness indices between the SNHGclusterA and SNHGclusterB. **(C)** The abundance of each immune signature in two SNHG interaction patterns. **(D)** GSEA reveals that SNHGclusterA is enriched in MES subtype and SNHGclusterB is enriched in PN subtype. **(E)** Alluvial diagram showing the changes of SNHG interaction patterns, GBM subtype, Geneclusters and SNHG scores. **(F)** Survival analyses for high SNHGscore and low SNHGscore GBM patients (P = 0.00022, Log-rank test). **(G)** Differences in SNHG scores among three GBM subtypes. **(H)** Differences in stemness indices between the high SNHGscore and low SNHGscore groups. **(I)** The abundance of each immune signature in the high SNHGscore and low SNHGscore groups. **(J)** IF staining was performed on GBM tissue sections of patients based on tissue sequencing (scale bar=15 μm). **(K)** The expression of MES/PN markers in high SNHGscore and low SNHGscore groups. All data are presented as the means ± SD, ns, P > 0.05, *P < 0.05, **P < 0.01, ***P < 0.001.

We further explored SNHGclusterA and SNHGclusterB in terms of subtype, stemness index and immune features based on the available experimental results and pathway analysis. SNHGclusterA had a higher proportion of CL subtype and MES subtype samples, while SNHGclusterB had a higher proportion of PN subtype samples (**Figure 4A**). GSEA also corroborated this finding: SNHGclusterA was significantly enriched for the MES subtype, whereas SNHGclusterB was enriched for the PN subtype (**Figure 4D**). Correlation analysis of the expression of PN/MES markers showed that SNHGclusterA exhibited higher expression of MES-type markers (**Figure S4C**). We calculated two indices of stemness, epigenetic features (mDNAsi) and gene expression (mRNAsi) based on the OCLR algorithm. EREG-mRNAsi (which reflects epigenetic regulation-related aspects of the mRNAsi) and EREG-mDNAsi (which reflects regulation-related aspects of the mDNAsi) parameters were also employed for comprehensive analysis (12). SNHGclusterB exhibited the dedifferentiation phenotype, while SNHGclusterA exhibited the differentiation phenotype (**Figure 4B**). For immune cells infiltration analysis, SNHGclusterA contained more suppressive immune features and had higher Treg infiltration, indicating that SNHGclusterA tended to be immunosuppressive subtype (**Figure 4C**). Additionally, we also assessed the correlation of each SNHG with immune features and the stemness index. In general, the SNHG family was negatively correlated with immunity and positively correlated with stemness (**Figures S4E, F**).

### Generation and functional annotation of SNHG-related gene sets

To further investigate the potential biological behavior of the two SNHG interaction patterns, we determined 168 SNHG-associated DEGs and performed unsupervised clustering analysis. We classified patients into three different genomic subtypes, which were named Gene.clusterA-C (**Figure S4D, Figure S5A**). **Figure S5F** lists the correlation of Gene.clusterA-C with the expression of each SNHG. Prognostic analysis showed that Gene.clusterB had the best prognosis, while Gene.clusterC exhibited the worst prognosis (**Figure S5B**). Similarly, we performed subtype, stemness index and immune cells infiltration analysis for each of these three genomic subtypes. Gene.clusterB exhibited the lowest percentage of the MES subtype, while Gene.clusterC exhibited the highest percentage of MES samples (**Figure S5C**). Correlation analysis of GBM subtype markers showed that Gene.clusterA and C had higher expression of MES-type markers, while Gene.clusterB had higher expression of PN-type markers (**Figure S5G**). From the stemness indices, Gene.clusterB had the strongest dedifferentiation phenotype and that Gene.clusterA and Gene.clusterC were biased toward the differentiated phenotype (**Figure S5D**). There was some similarity in immune feature trends and prognosis trends, as Gene.clusterC exhibited stronger immune activation features than Gene.clusterA and Gene.clusterB, and there was an even representation of immunostimulatory features and immunosuppressive features (**Figure S5E**).

### Construction and evaluation of SNHG scoring system

To assess individual SNHG interaction patterns, we constructed a scoring system, which we named SNHGscore, based on interaction pattern-related genes. Individual patient attribute changes were visualized with an alluvial diagram (**Figure 4E**). The relationship between SNHGscore and the expression of each SNHG related genes is shown in **Figure S6A**. A high SNHGscore was related to a poor prognosis, and a low SNHGscore was related to a favorable prognosis (**Figure 4F**). To evaluate the predictive efficiency of the SNHGscore, we performed time-ROC analysis. The predictive accuracy values of the SNHGscore for OS were 0.633, 0.699, 0.701, and 0.768 at 0.5, 1, 2, and 3 years, respectively (**Figure S6D**). The calibration curves for 0.5-, 1-, 2-, and 3-year OS predictions were approximate to ideal performance (**Figure S6E**). To quantify the risk of individual GBM patients, we produced a personalized score nomogram (**Figure S6F**). Time-ROC and calibration curve analyses were applied to evaluate the sensitivity and accuracy of the nomogram score, respectively (**Figures S6G, H**). Additionally, the SNHGscore distribution between two SNHGclusters and three gene.clusters were presented (**Figures S6B, C**). The MES subtype corresponded to a higher SNHGscore and the PN subtype corresponded to a lower SNHGscore (**Figure 4G**). The stemness indices showed that a low SNHGscore tended to correspond to the dedifferentiated state and a high SNHGscore tended to correspond to the differentiated state (**Figure 4H**). Immune feature analysis showed that a high SNHGscore represented high immune infiltration accompanied by high immunosuppression (**Figure 4I**). To further verify the utility of the SNHGscore system, we applied various public databases for iterative validation. The analysis based on the CGGA database was consistent with the previous analysis of the TCGA database (**Figures S7D–F**). We also observed that untreated patients with a high SNHGscore had a poor prognosis, while those with low SNHGscore and treated with chemotherapy or radiotherapy had a better prognosis (**Figures S7A–C**). Validation with the Rembrandt, Gravendeel, Frejie and Murat cohorts showed that a high SNHGscore was associated with a poor prognosis, while a low SNHGscore was associated with a favorable prognosis (**Figure S7G**).

We further assessed the RNA-sequencing data of tumor tissues from GBM patients in the Department of Neurosurgery, Qilu Hospital, Shandong University to calculate each patient’s SNHGscore (**Figure S7H**); The results showed that tumor tissues from patients with a high SNHGscore had lower SOX2 expression and higher CD44 expression, corresponding to the MES subtype. In contrast, tissue sections from patients with a low SNHGscore exhibited opposite results (**Figure 4J**). The correlation analysis between SNHGscore and the expression of PN/MES markers showed that a high SNHGscore corresponded to high expression of MES markers, while a low SNHGscore corresponded to high expression of PN markers (**Figure 4K**).

### The role of SNHG interaction patterns in anti-PD-1/L1 immunotherapy and chemotherapeutic drug selection

**Figure 5:**
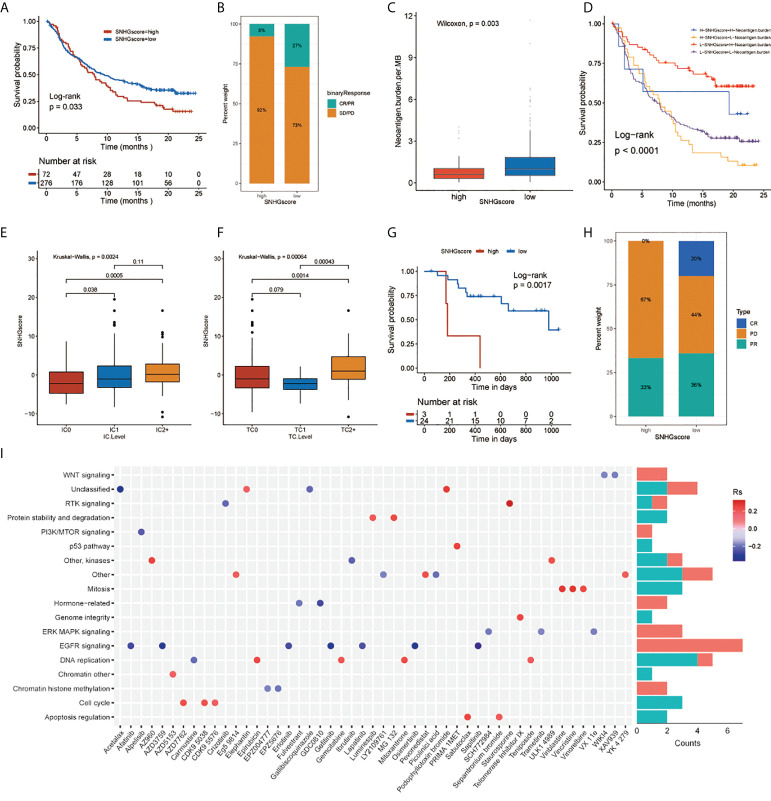
The relationship between SNHGscore and immunotherapy response and drug sensitivity. **(A)** Survival analyses for high and low SNHGscore patients in anti-PD-L1 immunotherapy cohort (P = 0.033). **(B)** Proportion of patients in the high or low SNHGscore group who responded to PD-L1 blockade immunotherapy. **(C)** Differences in neoantigen burden expression between high and low SNHGscore groups. (p = 0.003). **(D)** Survival analyses for patients receiving anti-PD-L1 immunotherapy stratified by both SNHGscore and neoantigen burden (P< 0.0001). **(E, F)** Differences in SNHGscore among three IC levels **(E)** and three TC levels **(F)**, respectively. **(G)** Survival analysis for high and low SNHGscore patient groups in the anti-PD1 immunotherapy cohort (GSE78220 cohort; P = 0.0017). **(H)** Proportion of patients in the high or low SNHGscore group who responded to PD-1 blockade immunotherapy. **(I)** Drug-targeted signaling pathways that are resistant or sensitive to SNHGscore.

We assessed the role of SNHGscore in the anti-PD-L1 cohort IMvigor210 and anti-PD-1 cohort GSE78220. Survival analysis showed that patients with low SNHG scores could benefit more from immunotherapy, showing a better prognosis (**Figures 5A, G**). Patients with low SNHG scores were more likely to respond favorably to anti-PD-1/L1 immunotherapy than patients with high SNHG scores (**Figures 5B, H**). Tumor neoantigen burden, closely linked to immunotherapeutic efficacy, was also assessed. Patients with low SNHGscore had higher neoantigen burden expression, suggesting a possible better efficacy against anti-PD-1/L1 immunotherapy (**Figure 5C**). Indeed, we found that patients with a combination of a low SNHG score and a high neoantigen burden showed a strong survival advantage (**Figure 5D**). The SNHGscore was significantly positively correlated with PD-L1 expression on immune cells (ICs), with IC0, IC1 and IC2+ corresponding to progressively higher SNHG scores (**Figure 5E**). Among tumor cells (TCs), the TC2+ group had a higher SNHGscore than the TC0 and TC1 groups (**Figure 5F**).

Next, we explored whether the SNHGscore system could be used as a predictor of antitumor drug sensitivity to guide clinical drug usage. A total of 49 drugs were identified by Spearman correlation analysis of SNHG scores with drug sensitivity (**Figure S7J**), (31). We also analyzed the signaling pathways of the genes targeted by these drugs (**Figure 5I**). The results showed that sensitizing drugs associated with a high SNHGscore mainly target the EGFR and ERK-MAPK signaling pathways. In contrast, the sensitizing drugs associated with low SNHG scores mainly target mitosis or cell cycle pathways.

### The MES GSC was more sensitive to AZD3759

**Figure 6:**
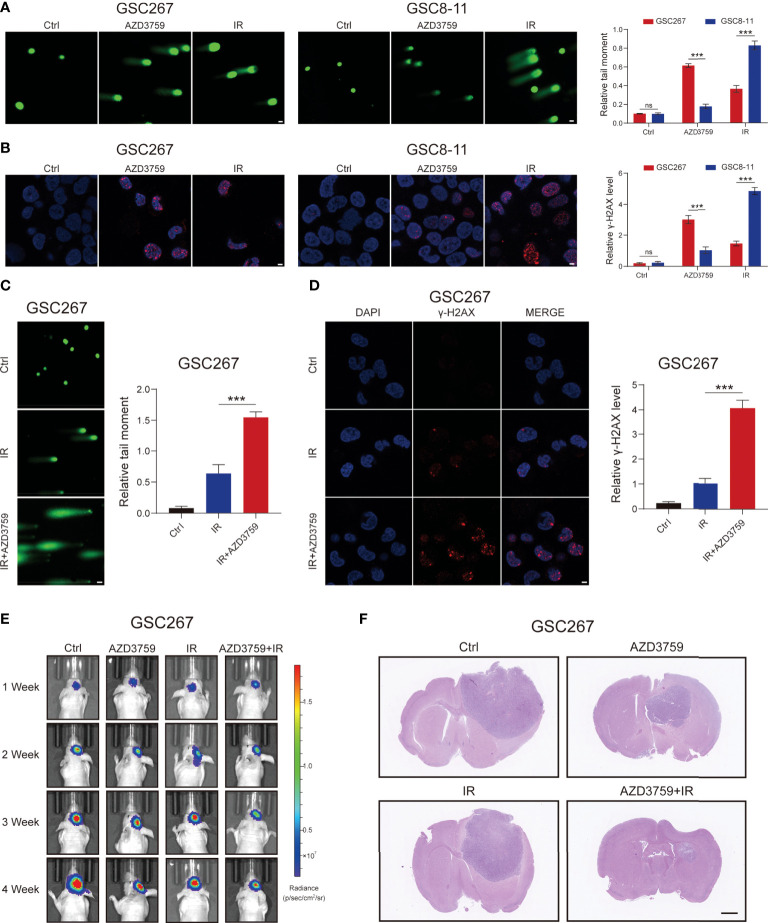
The MES GSC was more sensitive to AZD3759. **(A)** The comet assay showed the level of DNA damage in GSC267, and GSC8-11 treated with Zorifertinib or IR, respectively (scale bar=25 μm). The column plot represented the relative tail length. **(B)** Representative images and quantification of γ-H2A.X staining in GSC267 and GSC8-11 treated with Zorifertinib or IR, respectively (scale bar=15 μm). **(C, D)** comet assay **(C)** and IF assay**(D)** presented the DNA damage level in GSC267. **(E)** Bioluminescence imaging of tumor size of mice implanted with fluorescein-labeled GSC267. **(F)** H&E-stained brain sections of mice (scale bar=1 mm). All data are presented as the means ± SD, ns, P>0.05, ***P<0.001.

Based on the sequencing data of GSC cell lines at MD Anderson Cancer Center, we applied the SNHGscore system to score GSC cell lines and found that MES-type GSCs had high SNHG scores, while PN-type GSCs had low SNHG scores (**Figure S7I**). Subsequently, among the drugs analyzed above that were effective for cells with high SNHGscore, we selected the one that could cross the blood–brain barrier: AZD3759 (Synonyms: Zorifertinib), an EGFR inhibitor with excellent CNS permeability. AZD3759 is often used to study the treatment of brain metastases from lung cancer (32). The MES-type GSC 267 and PN-type GSC 8-11 were leveraged for experimental verification. The results of the comet assay and γ-H2AX IF staining showed that GSC 8 -11 was highly sensitive to radiotherapy and insensitive to AZD3759. In contrast, GSC267 cells were resistant to radiotherapy but sensitive to AZD3759 (**Figures 6A, B**). The combination of radiotherapy with AZD3759 significantly enhanced DNA damage in GSC 267 compared with that with radiotherapy alone (**Figures 6C, D**). This suggests that AZD3759 could enhance the sensitivity of GSC 267 to radiotherapy. Additionally, we applied the xenograft model to validate the conclusions drawn from the above fundings *in vivo*. Compared with radiation therapy or AZD3759 used alone, the combination of radiotherapy and AZD3759 significantly reduced tumor growth and prolonged the survival of the mice (**Figures 6E, F** and **Figure S6I**). We assumed that AZD3759 may affect the subtype of GSCs (in other words, initiating PMT) and lead to radiotherapy sensitization (11, 19). In summary, the SNHGscore system may be able to provide clinical guidance for GBM treatment selection.

### Predictive role of the SNHGscore system among pan-cancer

**Figure 7:**
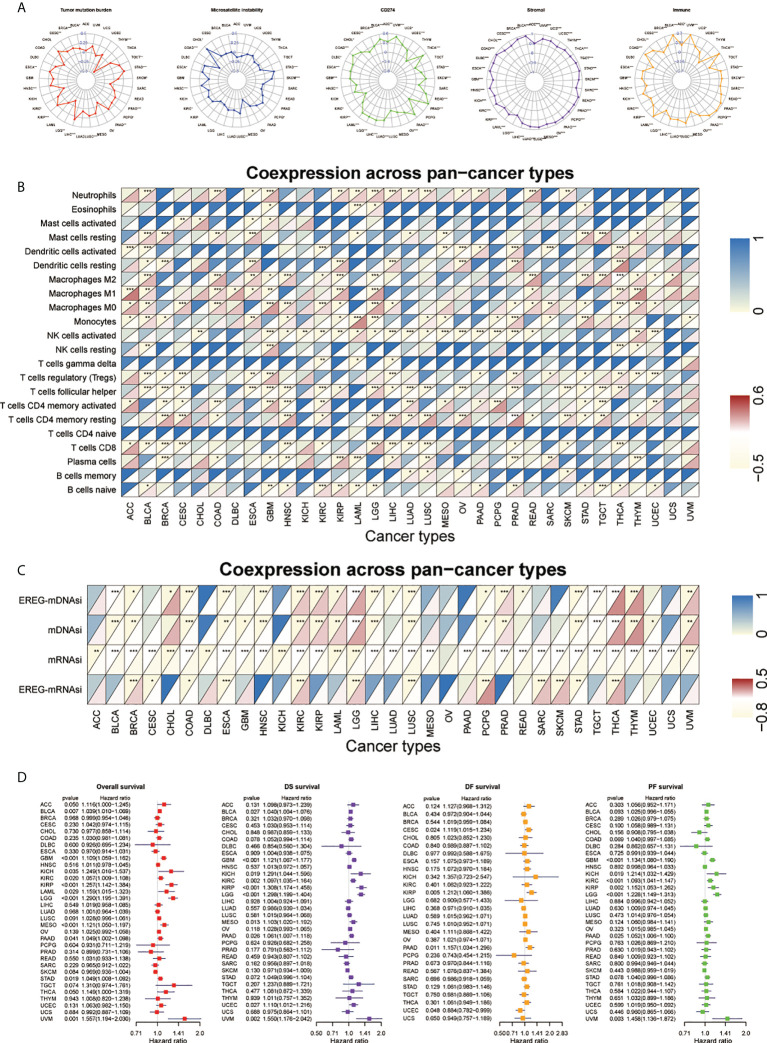
Performance of SNHGscore among pan-cancer. **(A)** Radar plot of the correlation between SNHGscore and TMB, MSI, CD274 expression, stromal score as well as immune score. **(B)** Relationship between SNHGscore and immune cell infiltration levels in pan-cancer. **(C)** Correlation between the SNHGscore and stemness indices among pan-cancer. **(D)** OS, DSS, DFS, and PFS analyses for the SNHGscore in pan-cancer calculated by univariate Cox regression algorithm. All data are presented as the means ± SD, ns, P>0.05, *P<0.05, **P<0.01, ***P<0.001.

We applied the SNHGscore system to other tumors to explore the role of the SNHGscore system among pan-cancer (**Table S5, S6**). First, we analyzed the predictive capacity of the SNHGscore for the immunotherapy response across cancers (**Figure 7A**). The radar chart showed that 17 of 33 cancers showed a significant correlation between the SNHGscore and TMB. MSI was significantly associated with SNHGscore in nine tumors. Correlation analysis of *CD274* and the SNHGscore showed significant relationships for up to 26 tumors, with 25 of them showing positive correlations. Analysis of stromal scores showed a strong positive correlation between the stromal score and SNHGscore for all 33 tumors. There were 29 cancers with immune scores that were strongly and positively correlated with the SNHGscore. The above results demonstrate the ability of our scoring system to accurately predict the response to immunotherapy. Next, we correlated the fractions of 22 immune cell types with the SNHGscore in each cancer (**Figure 7B**). We found that the fraction of immunosuppression-related immune cells was significantly associated with SNHGscore in most tumors. Of the 33 tumor types, all except ovarian serous cystadenocarcinoma (OV) showed a correlation of the stemness index with the SNHGscore. Nineteen cancers showed a negative correlation of the stemness index with SNHGscore, indicating that the trend in most tumors was consistent with that in our GBM study (**Figure 7C**). Finally, we analyzed the prognostic ability of the SNHGscore among pan-cancer (**Figure 7D**, **Figure S8**). The results showed that OS, disease-specific survival (DSS), disease-free survival (DFS) and progression-free survival (PFS) were positively correlated with SNHGscore in the vast majority of cancers (**Table S7**). Overall, the SNHGscore system performs similarly in predicting the prognosis and clinical features of patients with GBM and those with other tumors.

## Discussion

There is increasing evidence that SNHG plays an integral role in tumor development by interacting with various types of proteins and RNAs (28). Although there is a single study of SNHGs in the field of GBM, there is still a lack of research on the role of SNHGs in the subtype, immunological, and stemness features of GBM (33, 34). Exploring the role of different SNHG interaction patterns in GBM will help deepen our understanding of the mechanisms of GBM progression and guide more effective therapeutic strategies.

In this study, we first explored the relevance of the SNHG family in several malignant behaviors of GBM and found that SNHGs are closely related to the GBM subtype. We then screened *SNHG18* from 25 SNHGs as an entry point to validate its role in GSCs, showing that SNHG18 can affect the subtype and stemness of GSCs. From our assessment of 25 SNHGs, we demonstrated two SNHG interaction patterns: SNHGclusterA favors the MES subtype and differentiated phenotype, while SNHGclusterB favors the PN subtype and dedifferentiated phenotype. In terms of immune features, SNHGclusterA is strongly strong correlated with immunosuppressive cells such as Tregs, suggesting that patients in SNHGclusterA exhibit an immunosuppression phenotype (35, 36).

Furthermore, in our study, mRNA transcriptome differences between different SNHG interaction patterns were shown to be significantly correlated with biological pathways associated with SNHG and GBM features. These differentially expressed genes are considered SNHG-related signature genes. Similar to the clustering results of the SNHG interaction pattern, three genomic isoforms based on SNHG signature genes were identified and were also significantly associated with subtype, immune features, and stemness features of GBM. We developed a scoring system (SNHGscore) to assess the SNHG interaction patterns of individual GBM patients. SNHG interactions characterized by the MES subtype, differentiated phenotype, and immune infiltration with immunosuppression had a higher SNHGscore corresponding to a poorer prognosis. In contrast, a low SNHG score was associated with the opposite features and corresponded to a better prognosis. We assessed multiple databases, as well as our own patient-derived sequencing data and tissue section staining results, to demonstrate the accuracy of the SNHGscore. With the emergence of immune checkpoint blockade therapy, an increasing number of researchers are focusing on its use in the treatment of glioma (37, 38). The SNHGscore can be used to predict the sensitivity of patients to immunotherapy. MES-subtype glioma is generally resistant to treatments other than surgery (6, 39). We predicted dosing strategies for individuals with different scores; subsequently, we used the same GSC cell lines to experimentally validate the effects of AZD3759, which was among the drugs predicted by SNHGscore that could cross the blood–brain barrier (32, 40). MES-subtype GSC cell lines were sensitive to AZD3759, and its combination with radiotherapy significantly enhanced the damage to GSC cells. Finally, we attempted to apply the SNHGscore system to pan-cancer. Encouragingly, the SNHGscore does have a guiding role across cancers in terms of predicting immune and stemness features, the immunotherapy response and survival.

## Conclusions

In conclusion, our study confirmed the extensive regulatory mechanism of the SNHG family in GBM. Comprehensive evaluation of SNHG interaction patterns in individual tumors will help us to deepen our understanding of GBM subtype, immunity, and stemness. Additionally, it will guide more effective immunotherapy and radiotherapy-chemotherapy combination strategies to treat GBM.

## Data availability statement

The datasets presented in this study can be found in online repositories. The data presented in the study are deposited in the GEO repository, accession number GSE211554.

## Ethics statement

The research related to human use complied with all the relevant national regulations and institutional policies; the study was performed in accordance with the tenets of the Helsinki Declaration and was approved by the ethical committee of Qilu Hospital. The patients/participants provided their written informed consent to participate in this study. All animal experiments were approved by the Institutional Animal Care and Use Committee (IACUC) of Shandong University (Jinan, China). Written informed consent was obtained from the individual(s) for the publication of any potentially identifiable images or data included in this article.

## Author contributions

YF, ZG, XG, and GL designed this work. ZG, YF, JX, and HW integrated and analyzed the data. YF, ZG, and QG performed experiments. YF, ZG, and XG wrote this manuscript. ZG, YF, HX, RZ, and GL edited and revised the manuscript. All authors contributed to the article and approved the submitted version. The work reported in the paper has been performed by the authors unless clearly specified in the text.

## Funding

This work was supported by grants from the National Natural Science Foundation of China (Nos. 81874083; 82072776; 82072775; 81702468; 81802966; 81902540), Natural Science Foundation of Shandong Province of China (Nos. ZR2019BH057;ZR2020QH174; ZR2021LSW025), the Jinan Science and Technology Bureau of Shandong Province (2021GXRC029), Key clinical Research project of Clinical Research Center of Shandong University (2020SDUCRCA011) and Taishan Pandeng Scholar Program of Shandong Province (No. tspd20210322).

## Acknowledgments

We are grateful to Dr. Krishna P.L. Bhat for providing GSC cell lines used in our study. And we thank Dr. Yufeng Cheng and Jianzhen Wang of Department of Radiation Oncology at Qilu Hospital of Shandong University who helped us in radiation.

## Conflict of interest

The authors declare that the research was conducted in the absence of any commercial or financial relationships that could be construed as a potential conflict of interest.

## Publisher’s note

All claims expressed in this article are solely those of the authors and do not necessarily represent those of their affiliated organizations, or those of the publisher, the editors and the reviewers. Any product that may be evaluated in this article, or claim that may be made by its manufacturer, is not guaranteed or endorsed by the publisher.
