# Supplementary material for: Identification and validation of SNHG gene signature to predict malignant behaviors and therapeutic responses in glioblastoma
Source: Front Immunol. 2022 Sep 8;13:986615. doi: 10.3389/fimmu.2022.986615 (PMC9493242; doi:10.3389/fimmu.2022.986615)
Supplement: Supplementary file 1 [file DataSheet_1.pdf]

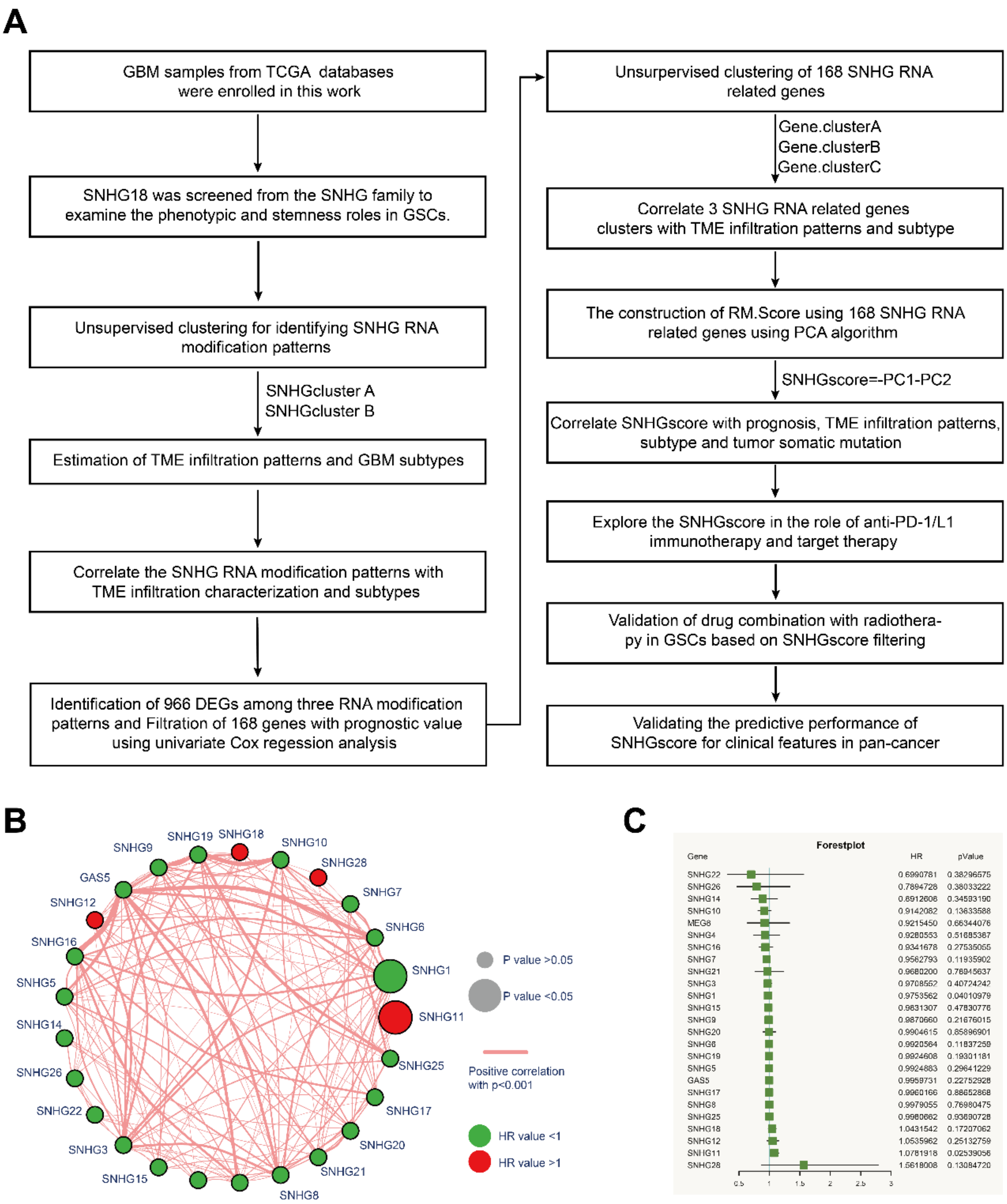

Figure S1

Figure S1 (A) Overview of the workflow. **(B)** The interaction between 25 SNHGs in TCGA-GBM. **(C)** Forest plot visualized the prognostic value of SNHGs in TCGA-GBM based univariate Cox analysis.

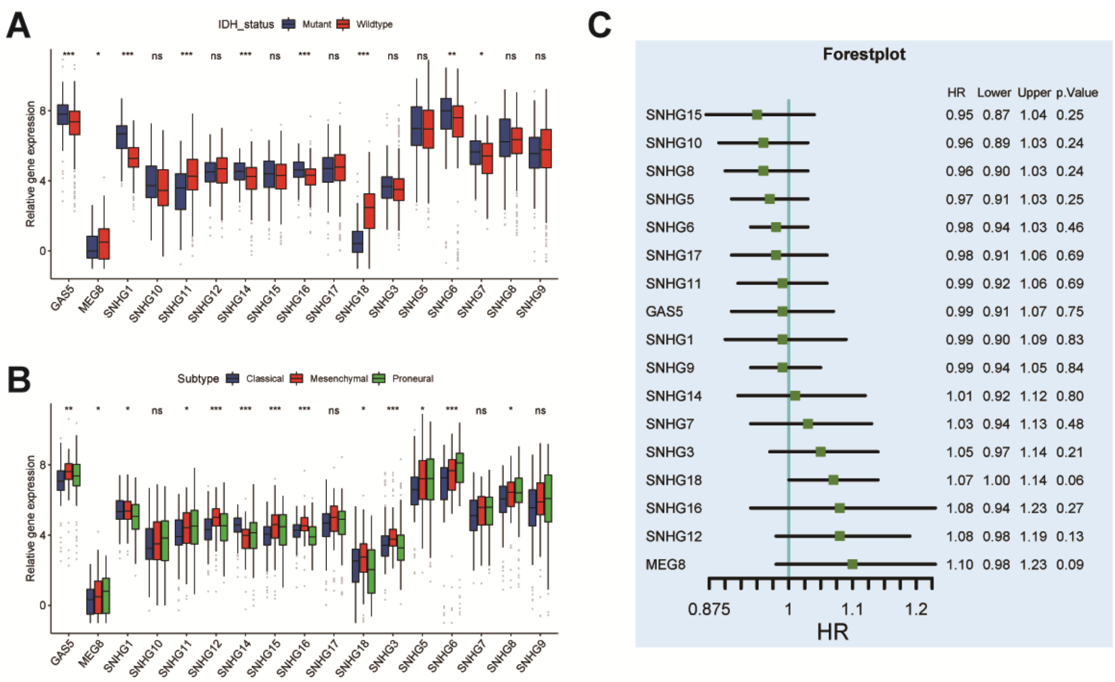

Figure S2

Figure S2 (A) The expression of 17 SNHGs between IDH mutant and IDH wild subtypes in CGGA. **(B)** The expression of 17 SNHGs between CGGA GBM subtypes. **(C)** Forest plot visualized the prognostic value of SNHGs in CGGA-GBM based univariate Cox analysis.

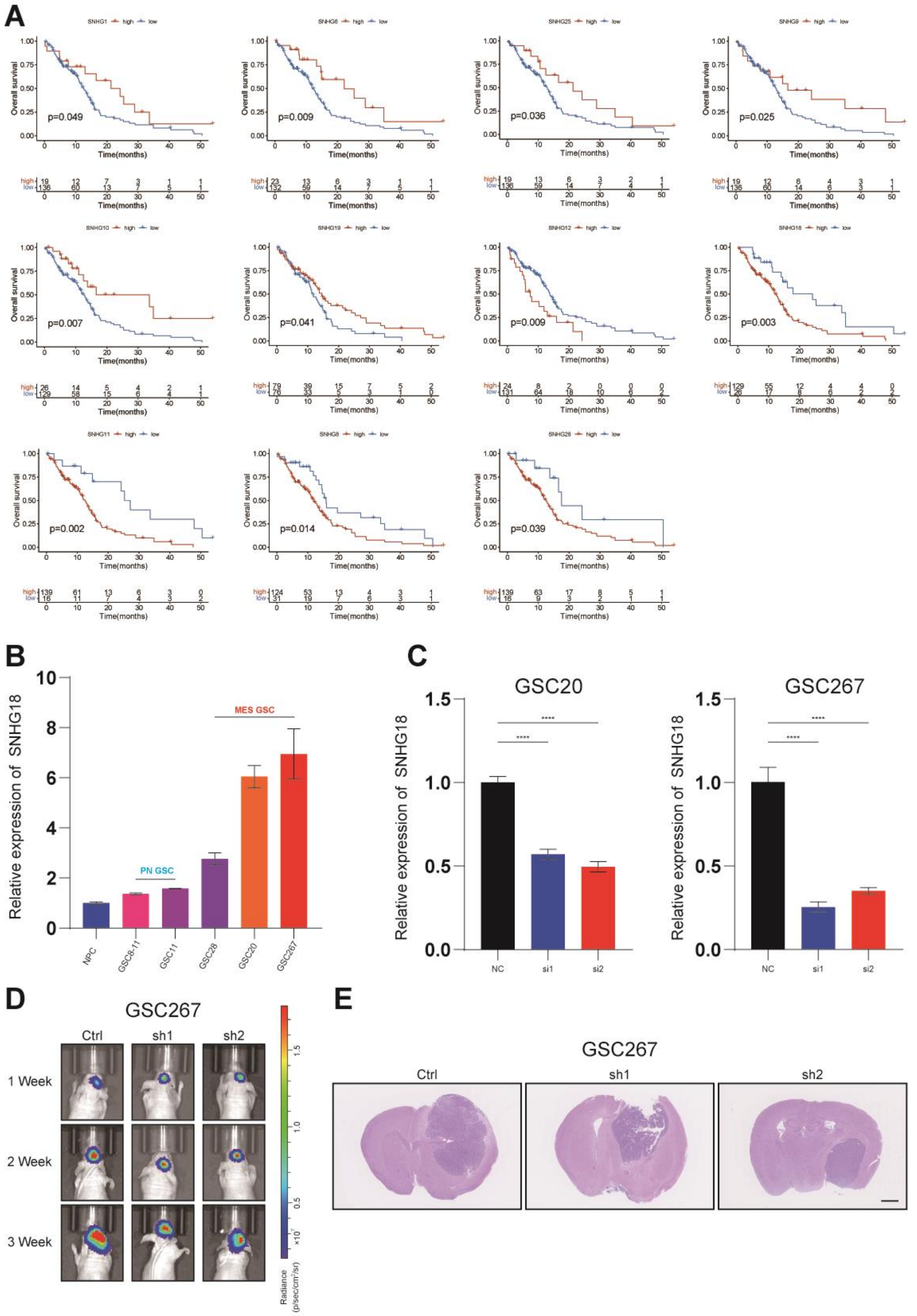

Figure S3

Figure S3 (A) KM curves showed 11 SNHGs with significant prognostic value in TCGA-GBMs. **(B)** The relative expression of SNHG18 among NPC and GSC cell lines. **(C)** The knockdown efficiency of SNHG18 in GSC20 and GSC267 respectively. **(D)** Bioluminescence imaging of tumor size of mice implanted with fluorescein-labeled

GSC267. **(E)** H&E-stained brain sections of mice (scale bar=1 mm).

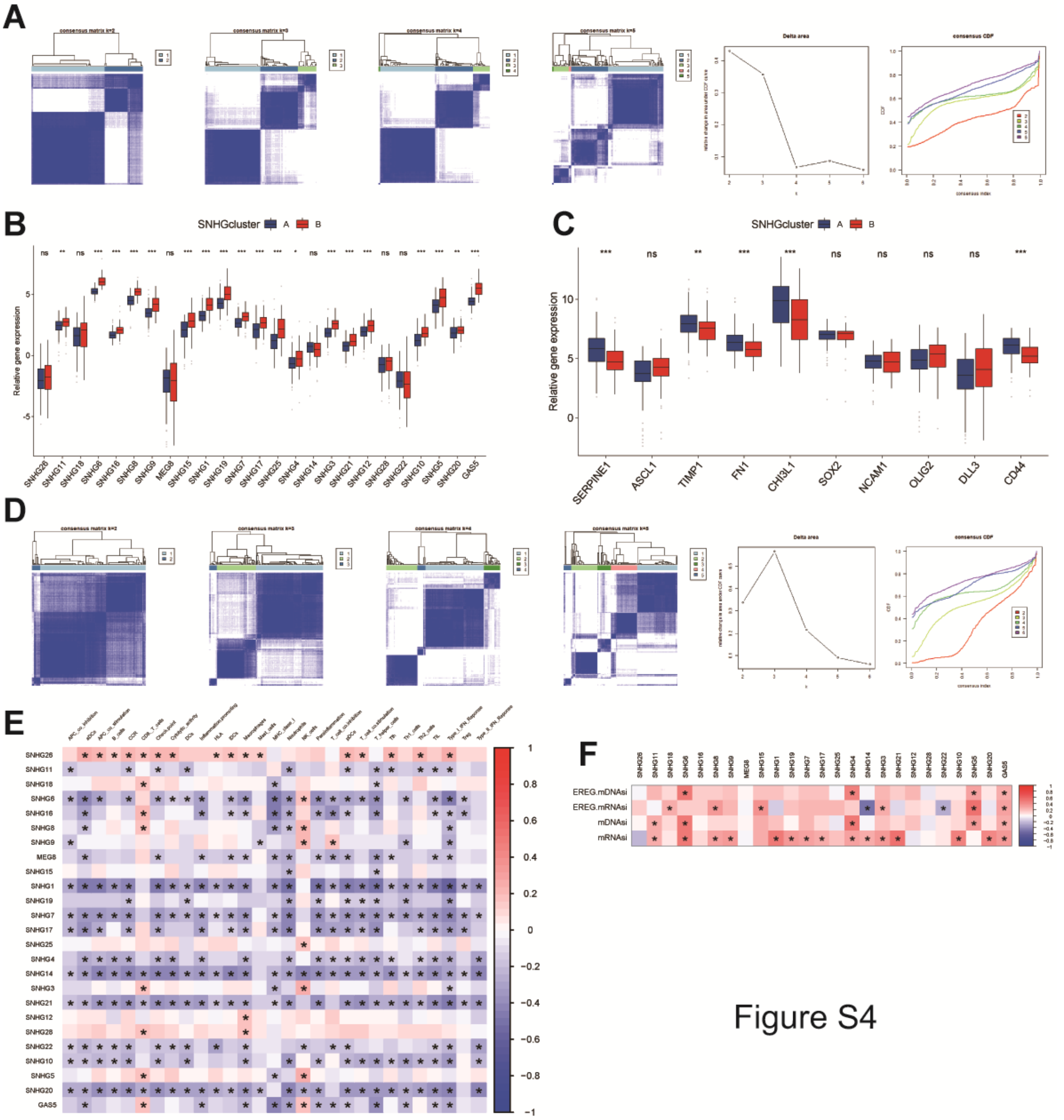

Figure S4

Figure S4 (A) Unsupervised clustering of 25 SNHGs in TCGA-GBM cohort and consensus matrices for k = 2 – 5. **(B)** The expression difference of 25 SNHGs among 2 SNHGclusters. **(C)** The expression difference of known MES and PN marker genes among 2 SNHGclusters. **(D)** Unsupervised clustering of SNHG related genes in TCGA-GBM cohort and consensus matrices for k = 2 – 5. **(E)** The correlation between each immune signature and each SNHG using spearman analyses. **(F)** The association between four stemness indices and the expression of SNHG genes.

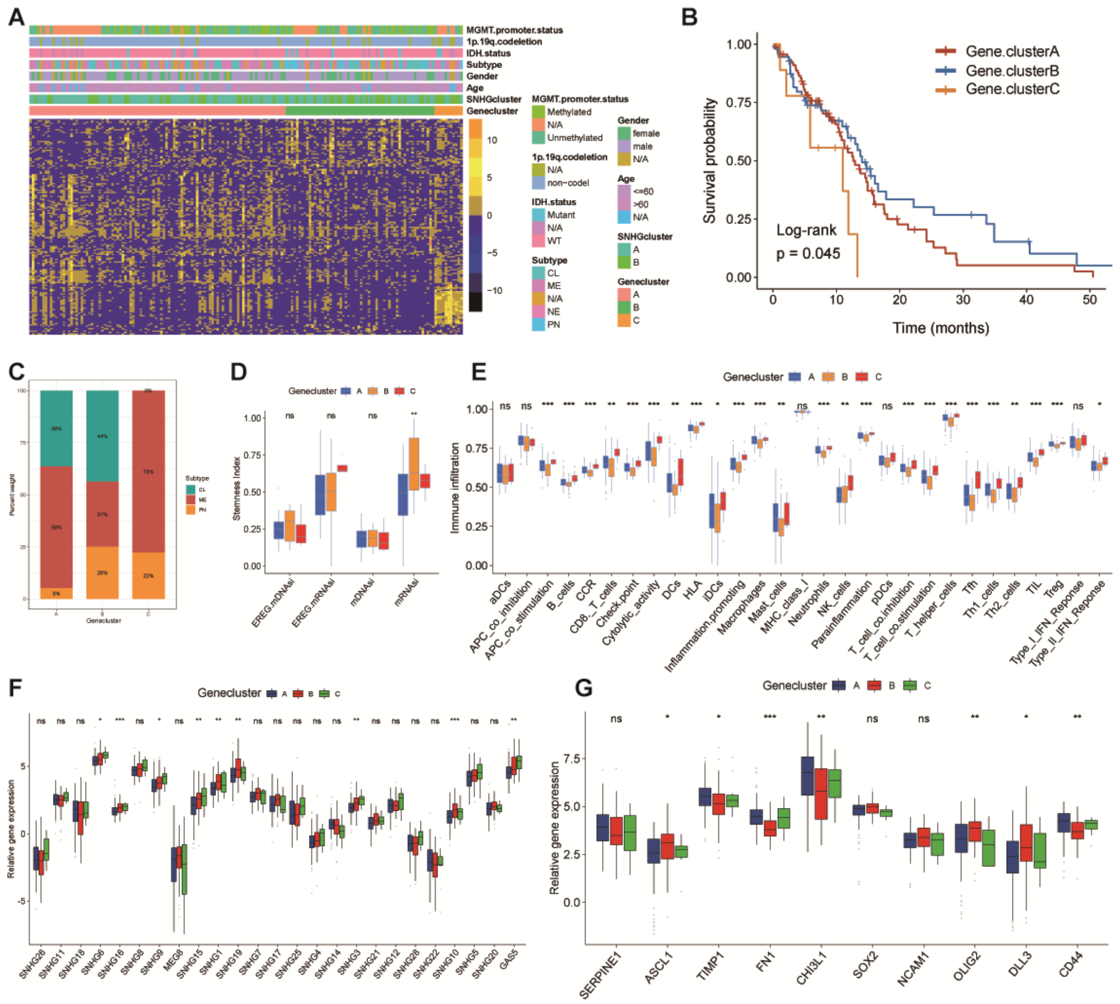

Figure S5

Figure S5 (A) Unsupervised clustering of 168 DEGs to divide patients into three gene.clusters, defined as Gene.clusterA, Gene.clusterB and Gene.clusterC, respectively. The Genecluster, SNHGcluster, age, gender, Subtype, IDH status, X1p.19q codeletion and MGMT status were annotated as clinical traits. **(B)** Survival analyses for Gene.clusterA, Gene.clusterB and Gene.clusterC patient groups using Kaplan-Meier curves. (P = 0.045, Log-rank test) **(C)** The proportion of MES, CL and PN subtypes among three Gene.clusters. **(D)** Differences in stemness indices between the GeneclusterA, GeneclusterB and GeneclusterC groups. **(E)** The abundance of each immune signature in the three Gene.clusters. **(F)** Difference in the 25 SNHGs expression between 3 geneclusters. **(G)** Difference in the known MES/PN marker genes expression between 3 geneclusters.

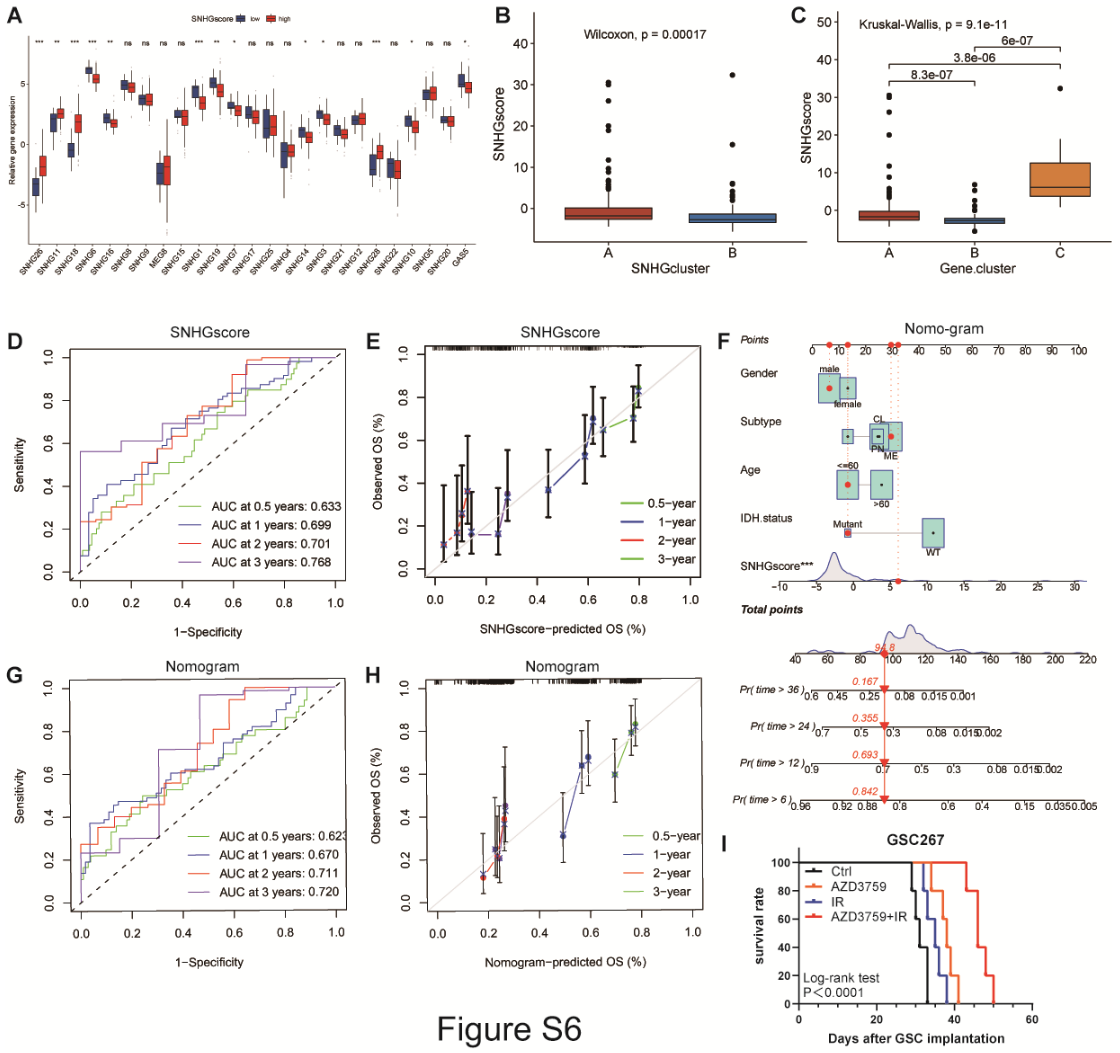

Figure S6

Figure S6 (A) The difference of SNHGs expression between low and high SNHGscore groups. **(B, C)** Differences in SNHG scores among two SNHG interaction patterns (B) and three Gene.clusters (C). **(D)** Time-dependent ROC analysis demonstrated that the survival prediction capacity of SNHGscore at 0.5, 1, 2, and 3-year. **(E)** Calibration curves showed the survival prediction of SNHGscore at 0.5, 1, 2, and 3-year. **(F)** Nomogram was constructed to predict 0.5, 1, 2, and 3-year OS probability with the SNHGscore and other clinical traits. **(G)** Time-dependent ROC analysis demonstrated that the survival prediction capacity of nomogram at 0.5, 1, 2, and 3-year. **(H)** Calibration curve of nomogram for predicting OS at 0.5, 1, 2, and 3-year. **(I)** Kaplan–Meier curves visualizing the survival of GSC267 xenograft mice in different groups.

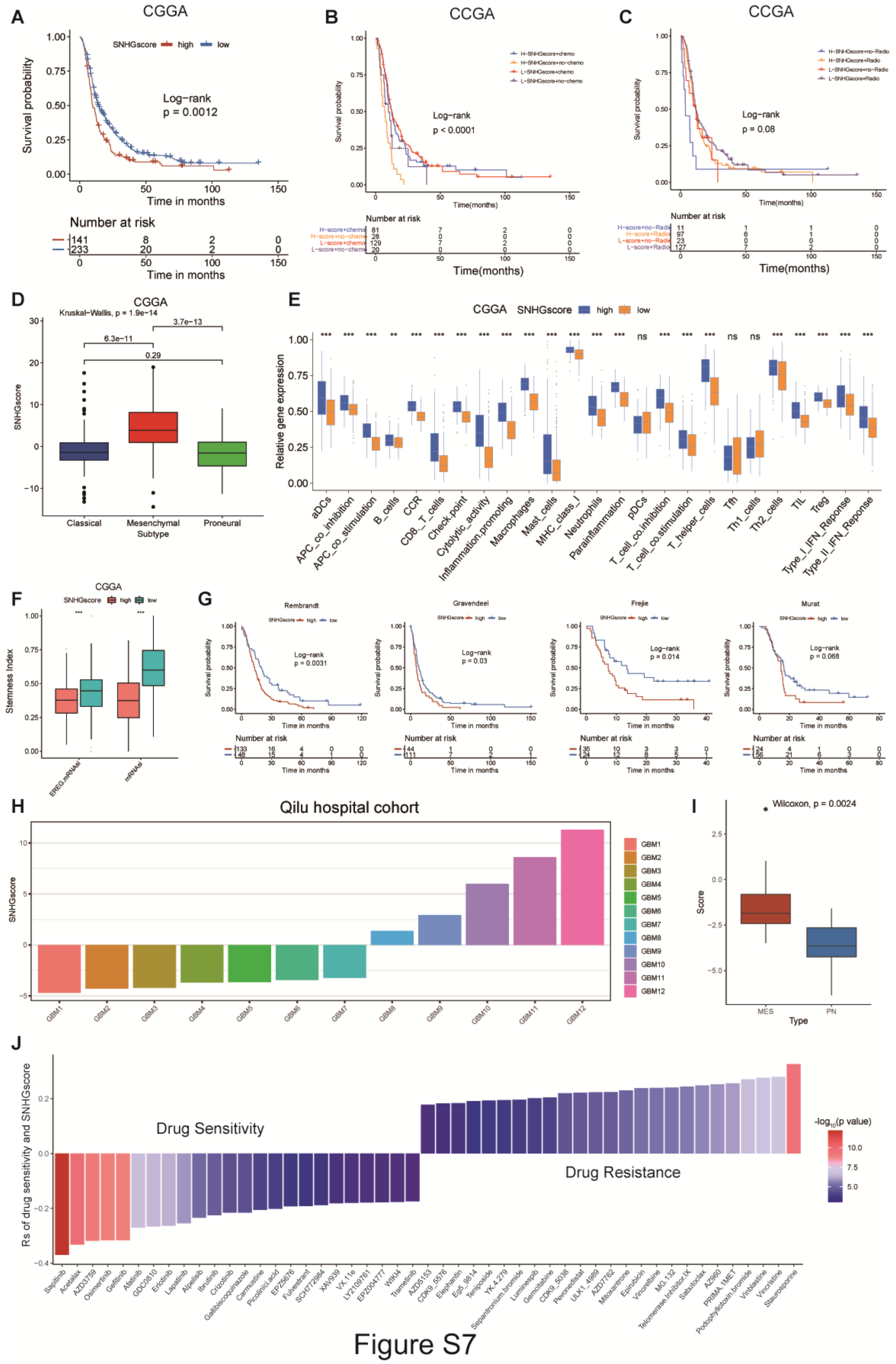

Figure S7

Figure S7 (A) Survival analyses for high SNHGscore and low SNHGscore CGGA GBM patients (P = 0.0012). **(B, C)** Survival analyses for subgroup patients stratified by both SNHGscore and treatment with chemotherapy or radiotherapy. (**D-F**) CGGA database was

used to verify the differences between high SNHGscore and low SNHGscore groups GBM subtype (D, P < 0.0001), immune signature (E) and stemness indices (F). **(G)** Survival analyses for high and low SNHGscore patient groups in the Rembrandt, Gravendeel, Frejie and Murat database using K-M curves (From left to right: P= 0.0031; P=0.03; P=0.014; P=0.068). **(H)** The SNHGscore of 12 GBM patients in Qilu hospital cohort. **(I)** The diffidence of SNHGscore between MES and PN subtype cell lines. **(J)** The correlation between SNHGscore and drug sensitivity calculated by the Spearman analysis.

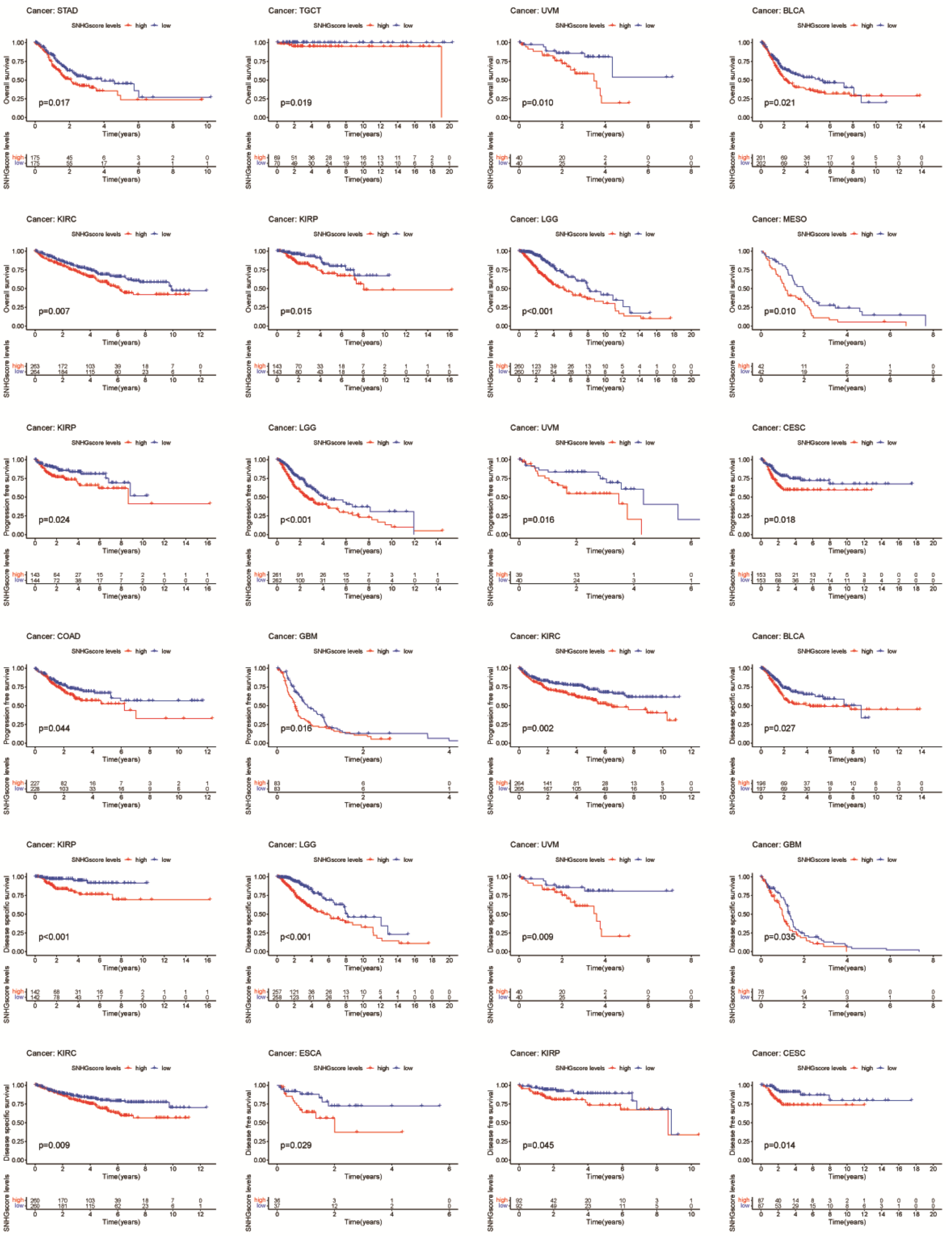

Figure S8

Figure S8 Kaplan-Meier analysis of the association between SNHGscore and OS, DFS, PFS, and DSS in pan-cancer.
